# 5-HT1A Receptor Agonist Promotes Retinal Ganglion Cell Function by Inhibiting OFF-Type Presynaptic Glutamatergic Activity in a Chronic Glaucoma Model

**DOI:** 10.3389/fncel.2019.00167

**Published:** 2019-05-03

**Authors:** Xujiao Zhou, Gang Li, Shenghai Zhang, Jihong Wu

**Affiliations:** ^1^Eye Institute, Eye and ENT Hospital, State Key Laboratory of Medical Neurobiology, Institutes of Brain Science and Collaborative Innovation Center for Brain Science, Shanghai Medical College, Fudan University, Shanghai, China; ^2^Shanghai Key Laboratory of Visual Impairment and Restoration, Shanghai, China; ^3^NHC Key Laboratory of Myopia (Fudan University), Key Laboratory of Myopia, Chinese Academy of Medical Sciences (Fudan University), Shanghai, China; ^4^Department of Ophthalmology and Vision Science, Eye and ENT Hospital, Fudan University, Shanghai, China

**Keywords:** 5-HT1A receptor, OFF-type RGCs, glaucoma, glutamate release, neuroprotection

## Abstract

Serotonin receptors are potential neuroprotective agents in degenerative diseases of the central nervous system. The protective effects of serotonin receptor (5-HT1A) agonists on the survival and function of retinal ganglion cells (RGCs) by regulating the release of the presynaptic neurotransmitter γ-aminobutyric acid (GABA) were confirmed in our previous study of a chronic glaucoma rat model. However, the roles of excitatory amino acids and their interactions with the 5-HT1A receptor in glaucoma remain unknown. Here, we found that ocular hypertension increased glutamine synthetase (GS) and excitatory amino acid transporter 2 (EAAT2) expression in rat retinas. In addition, the high expression of GS and EAAT2 induced by glaucoma was downregulated by the 5-HT1A receptor agonist 8-OH-DPAT and the 5-HT1A receptor antagonist WAY-100635, respectively. Patch-clamp techniques were used to record glutamate receptor-mediated spontaneous and miniature glutamatergic excitatory post-synaptic currents (sEPSCs and mEPSCs) as well as L-glutamate-induced current in OFF-type and ON-type RGCs in rat retinal slices. Although there were no significant differences in the frequency and amplitude of sEPSC and mEPSC release between normal and glaucoma OFF- and ON-type RGCs, exogenous 8-OH-DPAT administration specifically reduced the frequency, but not the amplitude, of sEPSC and mEPSC release in glaucoma OFF-type rather than ON-type RGCs; these effects were completely blocked by WAY-100635. In summary, 8-OH-DPAT decreases and increases GS and EAAT2 expression of glaucomatous retina, respectively, while decreasing sEPSC and mEPSC frequency. In contrast, WAY-100635 increases and decreases GS and EAAT2 expression of glaucomatous retina, respectively, while increasing sEPSC and mEPSC frequency. The reduction of glutamatergic presynaptic transmission by 8-OH-DPAT deactivates RGCs at the neural network level and reduces the excitotoxic damage in the pathological process of chronic glaucoma.

## Introduction

Serotonin [5-hydroxytryptamine (5-HT)] can be synthesized by many types of neurons in the central nervous system (CNS). The retina is the epitaxial part of the CNS. Both clinical and basic studies have found that serotonin is very important in the regulation of retinal ganglion cells (RGCs) (Hidaka, 2009). Convincing evidence demonstrates that serotonin also exists in the mammalian retina (Osborne et al., 1981; Chanut et al., 2002) and has many functions, including altering retinal amacrine cell processing; additionally, serotonin is expressed in the aqueous humor of the eyes, while 5-HT1A receptor agonist can reduce intraocular pressure (IOP) in rabbits, but not in monkeys (George et al., 2005; Sharif, 2010). In contrast, it has been found that activation of 5-HT7 receptors can increase the generation of aqueous humor, thus inducing the increase of IOP (Costagliola et al., 2008; Hsu et al., 2017). Serotonin can act on different receptors and regulate different functions, and according to its structure and function, it can be classified into seven families (5-HT1R to 5-HT7R), which are further subdivided into 14 subtypes. RT-PCR studies have revealed that the 5-HT1A, 5-HT2A, 5-HT2C, 5-HT2C, 5-HT3, and 5-HT7 receptor subtypes are expressed in the retinas of rats (Sharif and Senchyna, 2006), suggesting that the monoamine system plays an important role in the regulation of visual function.

The exchange of information between retinal neurons is mainly dependent on the chemical signals regulated by neurotransmitters located in the synaptic endings in the outer and inner retinal reticular layers (Yang, 2004). The survival of retinal neurons depends on the dynamic balance of excitatory and inhibitory neurotransmitter regulation systems (Murase, 2014). However, once this balance is broken, it leads to the occurrence of severe retinopathy (Kume et al., 2002; Akaike, 2006; Chang and Martin, 2009, 2011; Martin and Chang, 2012). Our prior studies have shown that activation of 5-HT1A receptors in the retina promotes RGC function by modulating presynaptic γ-aminobutyric acid (GABA) release in a chronic glaucoma model (Zhou et al., 2018). However, overactivation of glutamate receptors is known to cause excitotoxic damage to RGCs that leads to glaucoma (Moreno et al., 2005; Harada et al., 2007; Ishikawa et al., 2011; Almasieh et al., 2012; Bai et al., 2013). In the hippocampal CA1 region, 5-HT1A receptor-glutamate interactions, which reduce glutamate release from Schaffer collaterals to CA1 pyramidal neurons (Ciranna, 2006). In rat medullary dorsal horn neurons, the 5-HT1A receptor agonist 8-OH-DPAT reversibly decreases the amplitude of glutamatergic excitatory post-synaptic currents (EPSCs) and increases the paired-pulse ratio in a concentration-dependent manner (Choi et al., 2013). However, the roles of glutamatergic presynaptic inputs and their interactions with 5-HT1A receptors in chronic glaucoma remain unknown.

The functional characteristics of RGCs are determined by the cell body size, dendritic field size and dendrites of ganglion cell branches in the inner plexiform layer (IPL) (Morgan et al., 2008). Therefore, the locations at which dendritic lamellar bodies develop in the IPL largely determine the functional types of RGCs, as ON- and OFF-type RGC dendritic terminals terminate at different locations in the IPL layer. Therefore, to study the neural circuit changes related to glaucoma in more detail, the relationship between 5-HT1A receptors and glutamate release in ON- and OFF-type RGCs must be explored.

In our study, we used western blotting and patch-clamp recordings to explore whether the synthesis and transport of glutamate in the retina are affected by glaucoma, with a focus on glutamine synthetase (GS) and excitatory amino acid transporter 2 (EAAT2). In addition, we further investigated the synaptic modulatory effects of the 5-HT1A receptor agonist 8-OH-DPAT to understand the modulatory effects of serotonin in glaucoma.

## Materials and Methods

### Ethics Statement

This study was carried out in accordance with the recommendations of “the Association for Research in Vision and Ophthalmology (ARVO) Statement for the Use of Animals in Ophthalmic and Vision Research and the guidelines of Fudan University on the ethical use of animals.” The protocol was approved by the Fudan University committee. All measures were taken to minimize the suffering of animals and the number of animals used.

### Animals

A total of 190 adult male Wistar rats, aged 2 months and weighing 200 ± 10 g (SLAC Laboratory Animal, Co., Ltd., Shanghai, China), were used. The animals were kept under normal light conditions at 60–70% humidity with a 12 h light/dark cycle, and the ambient temperature was controlled at 23 ± 2°C. Intraperitoneal injections of ketamine and xylazine were administered to induce deep anesthesia.

### Rat Model of Ocular Hypertension

As previously described (Mittag et al., 2000; Wu et al., 2010; Chen et al., 2011; Zhou et al., 2018), the left eye was typically used as the control eye for the sham surgery, in which only three superior scleral veins were obtusely separated, but electrocoagulation was not performed. Three suprascleral veins were carefully isolated from the right eye and electrocoagulated at their branches. IOP measurements were made at 9 am daily, from 1 to 4 weeks after molding, using a calibrated tonometer (Tono-Pen XL; Mentor, Norwell, MA, United States), and five consecutive measurements were recorded for each eye to obtain the mean value (Mittag et al., 2000).

### Western Blotting

Briefly, retinal lysates were centrifuged at 12,000 ×*g* for 10 min at 4°C. Ten micrograms of each sample was separated by SDS-PAGE and electrotransferred to PVDF membranes (Immobilon-P; Millipore, Billerica, MA, United States). The membranes were blocked with non-fat milk (5%) for 1 h at room temperature and incubated with the following primary antibodies overnight at 4°C: a mouse monoclonal antibody against GS (ab64613, 1:1000; Abcam, Cambridge, MA, United States) and a rabbit monoclonal antibody against EAAT2 (ab205248, 1:1000; Abcam, Cambridge, MA, United States). The membranes were incubated with horseradish peroxidase-conjugated AffiniPure goat anti-mouse IgG (H + L) (115-035-003, 1:5000; Jackson ImmunoResearch Laboratories, West Grove, PA, United States) and horseradish peroxidase-conjugated AffiniPure goat anti-rabbit IgG (H + L) (111-035-003, 1:5000; Jackson ImmunoResearch Laboratories, West Grove, PA, United States). The relative intensities of the protein bands were quantified by scanning densitometry using ImageJ software (National Institutes of Health, Bethesda, MD, United States). GAPDH was used as an internal standard.

### Retinal Slice Preparation

Retinal slices were prepared as described previously (Zhou et al., 2017a,b, 2018). Following decapitation, the eyes were removed rapidly and transferred to ice-cold (4°C) artificial cerebrospinal fluid (ACSF) in which sucrose (124 mM) was substituted for NaCl. The components were as follows (in mM): sodium pyruvate 3, NaHCO_3_ 26, NaH_2_PO_4_ 1.25, sucrose 124, KCl 3, MgCl_2_ 3.8, CaCl_2_ 0.2, and glucose 10 (pH 7.4). The near-vitreous side of the retina was placed face-down on the filter paper and was cut using a manual slicer into 200-μm-thick slices for later use. These sections were incubated in oxygen-saturated ACSF before the cells were used in patch-clamp experiments (containing (in mM): 125 NaCl, 3 KCl, 26 NaHCO_3_, 1.25 NaH_2_PO_4_, 15 glucose, 2 CaCl_2_, and 1 MgCl_2_, pH 7.4), equilibrated with 95% O_2_ and 5% CO_2_.

### Whole-Cell Recording

The retinal slices were placed in a recording chamber, and oxygenated ACSF was continuously perfused at a rate of 2–3 ml/min. Interelectrode fluid with Lucifer yellow was injected into the RGC via a glass microelectrode, and the morphology of the RGC in the retinal section, including the position of the cell body and the deformation of the axon and dendrites, could be clearly distinguished by the combination of infrared differential interference contrast (IR-DIC) microscopy (Nikon, Tokyo, Japan) with a 40× magnification water-immersion objective lens (Li et al., 2017). Whole-cell spontaneous and miniature EPSCs (sEPSCs and mEPSCs, respectively) were recorded with an intracellular solution containing (in mM) CsMeSO_3_ 120, ATP-Mg 2, GTP-Na 0.2, EGTA 2, HEPES 10, NaCl 5, and TEA-Cl 10 (pH adjusted to 7.2 with CsOH; 275 mOsm/l). QX-314 (lidocaine *N*-ethyl bromide, 2.0 mM) was added to the pipette solution to block rapid Na^+^ currents.

### Data Analysis

The membranes of RGCs were sealed with a glass microelectrode with high resistance to form a whole cell model. An Axopatch Multiclamp 700B Amplifier (Axon Instruments, Foster City, CA, United States; sampling frequency 10 kHz, filter frequency 1 kHz) coupled to a Digidata 1440A digital analog converter system (Axon Instruments, Foster City, CA, United States) was used. The RGCs were injected at -70 mV. The sampling and filter frequencies were set at 10 and 1 kHz, respectively. Fast capacitance and slow capacitance compensation were employed through the 700B amplifier, and only the series resistance compensation was applicable (70–80%) (Ji et al., 2012). All spontaneous or miniature synaptic currents were detected using Clampfit 10.2 (Axon Instruments) and were analyzed with MiniAnalysis software (Synaptosoft, Fort Lee, NJ, United States). In the MiniAnalysis software parameter settings, the detection threshold for sEPSCs and mEPSCs was set to 5 pA to limit the inclusion of false-positive events (noise). Electrophysiological data were filtered using a low-pass elliptic filter with a cut-off frequency of 1 kHz.

### Drugs

Patch clamp experiments were conducted using a gravity perfusion system and a pressure injection system: the 5-HT1A receptor agonist 8-OH-DPAT [8-hydroxy-2-(di-*n*-propylamino) tetralin, 10 μM], the 5-HT1A receptor antagonist WAY-100635 (*N*-[2-[4-(2-methoxyphenyl)-1-piperazinyl] ethyl]-*N*-(2-pyridinyl), 10 μM). Tetrodotoxin was used to abolish spontaneous action potentials (TTX, 1 μM); other drugs applied included the ionotropic glutamate receptor antagonists 6-cyano-7-nitroquinoxaline-2,3-dione (CNQX, 10 μM) and D-2-amino-5-phosphonovalerate (AP5, 50 μM); the GABA_A_ receptor antagonist SR95531 (2-[3-carboxypropyl]-3-amino-6-(4-methoxyphenyl)-*p*yridazinium bromide, 10 μM), and the glycine receptor antagonist strychnine (5 μM). In some slices, WAY-100635 (10 μM) was applied beginning 10 min prior to 8-OH-DPAT application to block 5-HT1A receptors. For sEPSC recording, QX-314 (2.0 mM) was added to the pipette solution to block rapid Na^+^ currents. All drugs were purchased from Sigma-Aldrich.

### Statistical Analysis

Descriptive statistics are presented as the mean ± SEM. The mean frequency and amplitude before and after drug administration were statistically analyzed by Student’s *t*-test, and one-way analysis of variance (ANOVA) with Bonferroni’s *post hoc* test was performed for comparison of multiple groups using SPSS. The distributions of the amplitudes and the interevent intervals were compared using the Kolmogorov–Smirnov test. In all tests, *p <* 0.05 was considered statistically significant.

## Results

### High IOP-Induced Upregulation of GS Expression in Rat RGCs Could Be Reversed by 8-OH-DPAT

The preparation of the chronic glaucoma rat model followed the classical method reported by our laboratory to increase the IOP via scleral vein electrocoagulation (Wu et al., 2010; Zhou et al., 2017a,b, 2018). IOP increased from 2 to 4 weeks after model induction.

**FIGURE 1:**
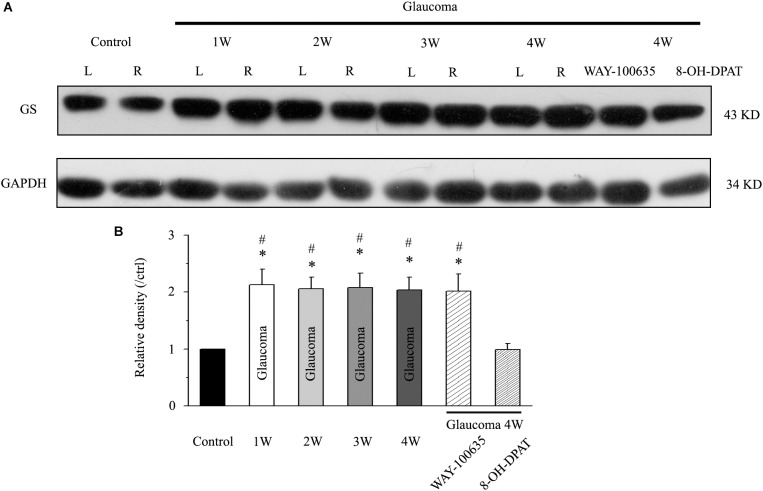
Chronic ocular hypertension and WAY-100635 upregulated GS expression in the rat retina, but this effect was reversed by 8-OH-DPAT. **(A)** Representative western blot of GS expression in control and glaucomatous retinas at 1, 2, 3, and 4 weeks after EVC and in glaucoma 4W + WAY-100635 retinas and glaucoma 4W + 8-OH-DPAT retinas. The expression of the 43 kDa protein was decreased from 1 week to up to 4 weeks after EVC. WAY-100635 upregulated GS expression and 8-OH-DPAT downregulated GS expression 4 weeks after glaucoma induction. **(B)** Densitometric analysis of GS expression from 1 to 4 weeks (*n* = 6) after EVC. GS expression was normalized to the expression in the control retinas. ANOVA, ^∗^*p* < 0.05 vs. control retina, #*p* < 0.05 vs. glaucoma 4W + 8-OH-DPAT. W, week; GS, glutamate synthetase; L, the left eye; R, the right eye.

We first examined whether there were differences in GS expression levels in the retinas of normal and glaucoma rats. Western blotting results showed that GS was highly expressed in the glaucoma retina compared with the normal retina in mice, from 1 to 4 weeks after electrocoagulation (Figure 1A). Antibodies against GS and GAPDH bound to single bands of approximately 43 and 34 kDa, respectively. The GS protein level increased to 213 ± 27% of the normal group at 1 week, to 206 ± 20% of the normal group at 2 weeks, to 208 ± 25% of the normal group at 3 weeks and to 204 ± 22% of the normal group at 4 weeks after high IOP (*n* = 6, *p* < 0.05; Figure 1B). In glaucomatous rats, intravitreal injection of the 5-HT1A receptor agonist 8-OH-DPAT significantly attenuated the high IOP-induced elevations in retinal GS protein expression to 99 ± 11% of the normal group (*n* = 6, *p* < 0.05; Figure 1B). In contrast, intravitreal injection of the 5-HT1A receptor antagonist WAY-100635 did not attenuate the high IOP-induced increases in GS expression in glaucomatous rats; the expression levels remained at 202 ± 30% of the control group levels (*n* = 6, *p* < 0.05; Figure 1B). These findings indicate that stimulation of 5-HT1A receptors in the retina attenuates glaucoma-induced increases in GS protein expression.

### High IOP-Induced Increases in EAAT2 Expression Were Reversed by a 5-HT1A Receptor Antagonist

**FIGURE 2:**
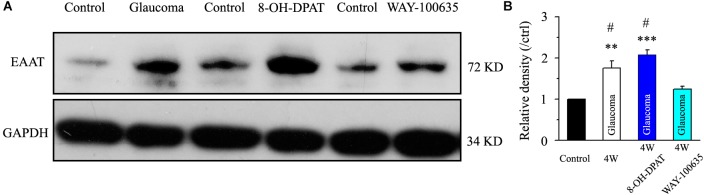
Chronic ocular hypertension and 8-OH-DPAT upregulated EAAT2 expression in the rat retina, but this effect was reversed by WAY-100635. **(A)** Representative western blot of EAAT2 expression in control and glaucomatous retinas 4 weeks after EVC and in glaucoma 4W + 8-OH-DPAT retinas and glaucoma 4W + WAY-100635 retinas. The expression of the 72 kDa protein was increased in the glaucomatous retinas and in the glaucoma 4W + 8-OH-DPAT group. WAY-100635 downregulated EAAT2 expression 4 weeks after glaucoma induction. **(B)** Densitometric analysis of EAAT2 expression in the glaucoma 4W and drug intervention groups (*n* = 7). EAAT2 expression was normalized to the expression in the control retinas. ANOVA, ^∗∗^*p* < 0.01 and ^∗∗∗^*p* < 0.001 vs. control retina, #*p* < 0.05 vs. glaucoma 4W + WAY-100635. W, week; EAAT, glutamate transporter.

**FIGURE 3:**
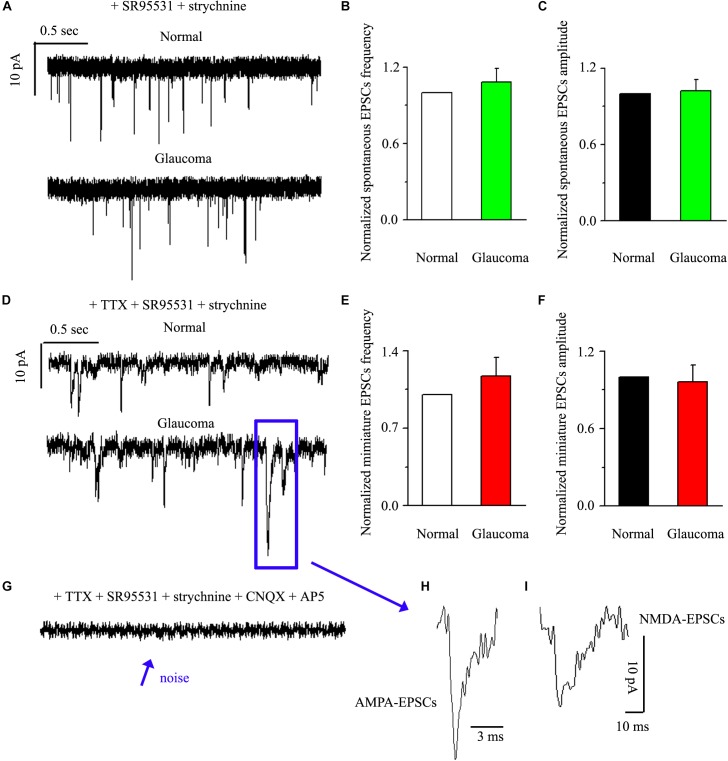
Ocular hypertension did not significantly change the frequency or amplitude of sEPSCs and mEPSCs in RGCs. **(A)** Top: representative recording from a normal rat experiment. Vertical scale bar: 10 pA; horizontal scale bar: 0.5 s. Bottom: representative recording from a glaucomatous rat experiment. Vertical scale bar: 10 pA; horizontal scale bar: 0.5 s. Summarized data for the frequency **(B)** and amplitude **(C)** of sEPSCs between normal and glaucomatous RGCs (*n* = 7). **(D)** Top: representative recording from a normal rat experiment. Vertical scale bar: 10 pA; horizontal scale bar: 0.5 s. Bottom: representative recording from a glaucomatous rat experiment. Vertical scale bar: 10 pA; horizontal scale bar: 0.5 s. Summarized data for the frequency **(E)** and amplitude **(F)** of mEPSCs between normal and glaucomatous RGCs (*n* = 9). **(G)** The signal and noise can be identified under recording conditions in which TTX + SR95531 + strychnine + CNQX +AP5 are administered. **(H)** The enlarged AMPA component EPSCs in the blue box of **(D)**; horizontal scale bar: 3 ms. **(I)** The enlarged NMDA component EPSCs in the blue box of **(D)**; vertical scale bar: 10 pA; horizontal scale bar: 10 ms. The results in **(B,C,E,F)** are expressed as the mean ± standard error.

Next, we tested the expression levels of EAAT2 before and after glaucoma model induction and drug intervention. The results showed that EAAT2 upregulation could be detected in the retinas of glaucoma and in the retinas of glaucoma following vitreous injection of 8-OH-DPAT (Figure 2A). However, WAY-100635 injection into the vitreous cavity of glaucomatous rats attenuated the glaucoma-induced increase in EAAT2 (Figure 2A). EAAT2 levels in the glaucoma group increased to 176 ± 17% of those in the control group (*n* = 7, *p* < 0.01; Figure 2B), the levels in the glaucoma + 8-OH-DPAT group increased to 208 ± 12% of those in the control group (*n* = 7, *p* < 0.001; Figure 2B), and the levels in the glaucoma + WAY-100635 group increased to only 125 ± 6% of those in the control group (*n* = 7, *p* > 0.05; Figure 2B). The effect of 8-OH-DPAT or WAY-100635 on the expression of GS and EAAT2 protein was detected after injection into the vitreous cavity of normal rats, as shown in the Supplementary Figure S1. These findings indicate that stimulation of 5-HT1A receptors in the glaucomatous retina increases EAAT2 protein expression and that inhibition of 5-HT1A receptors in the glaucomatous retina reduces glaucoma-induced increases in EAAT2 protein expression. Since 5-HT1A receptors can regulate GS and EAAT2, we next examined whether 5-HT1A receptors can affect the synaptic afference of the neurotransmitter glutamate. The glutamatergic sEPSCs were isolated by inclusion of SR95531 (10 μM) and strychnine (5 μM) in the bath to prevent GABA and glycine receptors, respectively. In some experiments, TTX (1 μM) was included in the bath to block action potential generation and polysynaptic action. Figure 3A,D illustrate sweeps of sEPSCs and mEPSCs from control and glaucomatous rat RGCs, showing that the mean sEPSC and mEPSC frequency and amplitude were not significantly different (Figure 3B: the sEPSC frequency in the glaucomatous RGC was 108 ± 11% of that in the control RGC; Figure 3C: the sEPSC amplitude in the glaucomatous RGC was 102 ± 9% of that in the control RGC; *n* = 7, *p* > 0.05. Figure 3E: the mEPSC frequency in the glaucomatous RGC was 117 ± 17% of that in the control RGC; Figure 3F: the mEPSC amplitude in the glaucomatous RGC was 96 ± 13% of that in the control RGC; *n* = 9, *p* > 0.05). These results indicate that the frequency and amplitude of glutamatergic sEPSCs and mEPSCs were not significantly different between the control and glaucomatous RGCs. At the end of the experiment, we confirmed blockage of the synaptic currents by adding a trace recorded in the presence of CNQX (10 μM) and AP5 (50 μM) (Figure 3G) to help visualize the signal-to-noise ratio and the glutamatergic nature of the EPSCs (Figure 3H,I). The rapid AMPA component had a rise time of only 200 μs and a decay time constant of only 1–3 ms; with slow NMDA components, the peak value was reached within approximately 10 ms, and the attenuation constants were 50 and 250 ms.

### 8-OH-DPAT Significantly Reduced the Frequency, but Not the Amplitude, of Glutamatergic sEPSCs in OFF-Type RGCs in Glaucoma

**FIGURE 4:**
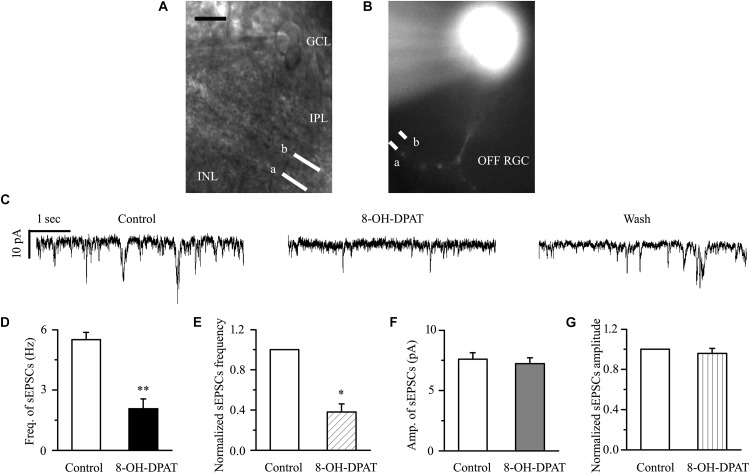
8-OH-DPAT significantly reduced the frequency, but not the amplitude, of glutamatergic sEPSCs in OFF-type RGCs. **(A,B)** The micrograph in **(A)** was taken with an infrared interferometric phase microscope, and the retinal layers can be seen. Representative Lucifer yellow-filled OFF-type RGCs with dendritic arborizations in the proximal (a) and distal (b) parts of the IPL are shown. GCL, ganglion cell layer; IPL, inner plexiform layer; INL, inner nuclear layer. Scale bar: 10 μm. **(C)** Representative traces showing the effect of 10 μM 8-OH-DPAT on glutamatergic sEPSCs. Vertical scale bar: 10 pA; horizontal scale bar: 1 s. **(D)** Summarized data from 11 RGCs showing that 10 μM 8-OH-DPAT significantly decreased the average frequency of glutamatergic sEPSCs. **(E)** Normalized glutamatergic sEPSC frequency (*n* = 11). **(F)** Summarized data from 11 RGCs showing that the average amplitude of glutamatergic sEPSCs was not significantly changed by 10 μM 8-OH-DPAT. **(G)** Normalized glutamatergic sEPSC amplitude (*n* = 11). ^∗^*p* < 0.05 and ^∗∗^*p* < 0.01 vs. control, Student’s paired *t-*test.

There are many types of RGCs in the retina. This study mainly focuses on two categories of RGCs – ON- (*n* = 18) and OFF-type RGCs (*n* = 43) – and the effect of 8-OH-DPAT on local glutamatergic afferents of RGCs was detected. The ON-type RGCs (accounting for 30%) and OFF-type RGCs (accounting for 70%) were identified according to well-established morphological and physiological indicators (Famiglietti and Kolb, 1976; Margolis and Detwiler, 2007). Morphologically, ON- and OFF-type RGCs were characterized based on whether their dendrites terminate in the proximal (a) or distal (b) parts of the IPL (Figure 4A,B, 9A,B). We first tested OFF-type RGCs. Based on the local glutamatergic bipolar neurons projecting onto RGCs, we next assessed the role of 5-HT1A receptor agonist in local glutamatergic inputs to RGCs (sEPSCs). Application of 8-OH-DPAT (10 μM) did not alter the amplitude but significantly decreased the frequency of the glutamatergic sEPSCs (Figure 4C) in glaucomatous RGCs. The frequency decreased from the normal baseline of 5.51 ± 0.36 to 2.07 ± 0.49 Hz in the drug intervention group (*n* = 11, *p* < 0.001; Figure 4D), equivalent to 38 ± 8% of the normal group (*n* = 11, *p* < 0.01; Figure 4E). The amplitude of the glutamatergic sEPSCs before and during 8-OH-DPAT application was 7.59 ± 0.54 and 7.23 ± 0.48 pA, respectively (*n* = 11, *p* > 0.05; Figure 4F), equivalent to 96 ± 5% of the normal group (*n* = 11, *p* > 0.05; Figure 4G). This effect is most noticeable after approximately 5 min of treatment, 10 min for maximum effect, followed by 20 to 30 min of fresh extracellular fluid irrigation for complete recovery to baseline. At the end of the experiments, 10 μM CNQX and 50 μM AP5 blocked all of the glutamatergic sEPSCs.

### WAY-100635 Prevented the Inhibitory Effects of 8-OH-DPAT on Glutamatergic sEPSCs in OFF-Type RGCs in Glaucoma

**FIGURE 5:**
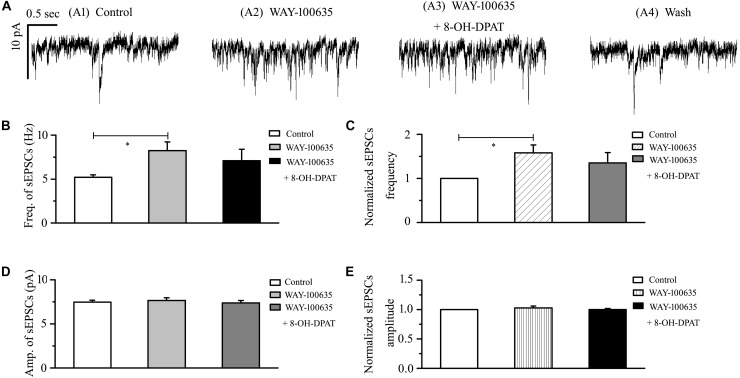
WAY-100635 prevented the inhibitory effects of 8-OH-DPAT on sEPSCs in OFF-type RGCs. **(A)** Representative recordings during control conditions **(A1)**, during WAY-100635 (10 μM) application **(A2)**, during WAY-100635 (10 μM) + 8-OH-DPAT (10 μM) application **(A3)** and during recovery **(A4)**. Vertical scale bar: 10 pA; horizontal scale bar: 0.5 s. After preincubation with WAY-100635 (10 μM), 8-OH-DPAT (10 μM) did not significantly change either the frequency **(B)** or amplitude **(D)** of EPSCs in RGCs (*n* = 8). **(C,E)** Normalized glutamatergic sEPSC frequency (*n* = 8) and amplitude. Note that WAY-100635 increased the baseline frequency **(B,C)** of the sEPSCs. ^∗^*p* < 0.05, ANOVA.

In glaucomatous RGCs, the responses of glutamatergic sEPSCs to 8-OH-DPAT were sensitive to the 5-HT1A receptor antagonist WAY-100635. In the presence of WAY-100635 (10 μM), 8-OH-DPAT (10 μM) did not significantly change the frequency (5.21 ± 0.26 Hz before vs. 7.08 ± 1.32 Hz after 8-OH-DPAT application; *n* = 8, *p* > 0.05; Figure 5B) of glutamatergic sEPSCs (Figure 5A), which remained at 135 ± 24% of the control level (*n* = 8, *p* > 0.05; Figure 5C). Similarly, in the presence of WAY-100635 (10 μM), 8-OH-DPAT (10 μM) did not significantly change the amplitude (7.46 ± 0.23 pA before vs. 7.38 ± 0.28 pA after 8-OH-DPAT application; *n* = 8, *p* > 0.05; Figure 5D) of glutamatergic sEPSCs, which remained at 100 ± 2% of the control level (*n* = 8, *p* > 0.05; Figure 5E). As shown in Figure 5B,C, WAY-100635 alone markedly increased the baseline frequency, but not the amplitude, of glutamatergic sEPSCs. The sEPSC frequency was 5.21 ± 0.26 Hz in control groups and 8.23 ± 1.01 Hz in WAY-100635-treated groups (*n* = 8, *p* < 0.05; Figure 5B), 158 ± 18% of the control level (*n* = 8, *p* < 0.05; Figure 5C).

### 8-OH-DPAT Decreased mEPSCs in OFF-Type RGCs in Glaucoma

**FIGURE 6:**
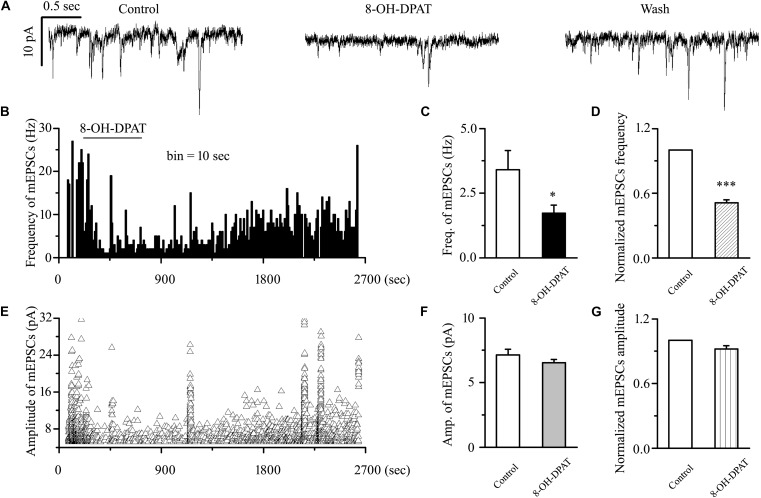
8-OH-DPAT significantly decreased the frequency, but not the amplitude, of mEPSCs in OFF-type RGCs. **(A)** Representative traces showing the effect of 10 μM 8-OH-DPAT on mEPSCs. Vertical scale bar: 10 pA; horizontal scale bar: 0.5 s. **(B,E)** Frequency (10 s bin) and amplitude histograms of the mEPSCs from the trace in **(A)** showing the effects of 8-OH-DPAT. Summarized data from 11 RGCs showing that 10 μM 8-OH-DPAT significantly decreased the average frequency **(C)** and amplitude **(F)** of the mEPSCs. **(D,G)** Normalized mEPSC frequency and amplitude (*n* = 11). ^∗^*p* < 0.05 and ^∗∗∗^*p* < 0.001 vs. control, Student’s paired *t-*test.

sEPSC includes components of mEPSC and other components. There are two types of action potential: dependent and non-dependent. However, whether the effect of 8-OH-DPAT depended on the action potential remained unknown. The release of neurotransmitters at the synapse terminal can be directly regulated, allowing mEPSCs to be tracked. In our experimental condition, the frequency of mEPSC was significantly less than that of sEPSC, but there was no difference in amplitude between the two. The frequency of sEPSCs was 5.51 ± 0.36 Hz, and the frequency of mEPSCs was 3.41 ± 0.74 Hz (*n* = 11, *p* < 0.01). In further assessments of the effect of the 5-HT1A receptor on mEPSCs in OFF-type RGCs in glaucomatous RGCs, 8-OH-DPAT (10 μM) significantly decreased the frequency, but not the amplitude, of the mEPSCs (Figure 6A). The frequency histogram (Figure 6B) and the graph of the running amplitude (Figure 6E) of glutamatergic mEPSCs in a representative OFF-type RGC show the time course of the frequency and amplitude response to 8-OH-DPAT application. The frequency decreased from 3.41 ± 0.74 to 1.72 ± 0.32 Hz (*n* = 11, *p* < 0.05; Figure 6C), 51 ± 3% of the control level (*n* = 11, *p* < 0.001; Figure 6D). The amplitude did not decrease (7.13 ± 0.44 pA in the control group vs. 6.54 ± 0.26 pA in the treated group; *n* = 11, *p* > 0.05; Figure 6F), remaining at 92 ± 3% of the control level (*n* = 11, *p* > 0.05; Figure 6G). The 8-OH-DPAT-induced responses started within 5 min and were reversible. At the end of the experiments, application of 10 μM CNQX and 50 μM AP5 abolished all of the mEPSCs.

### 8-OH-DPAT-Induced Decreases in the Frequency of mEPSCs in OFF-Type RGCs in Glaucoma Were Blocked by WAY-100635

**FIGURE 7:**
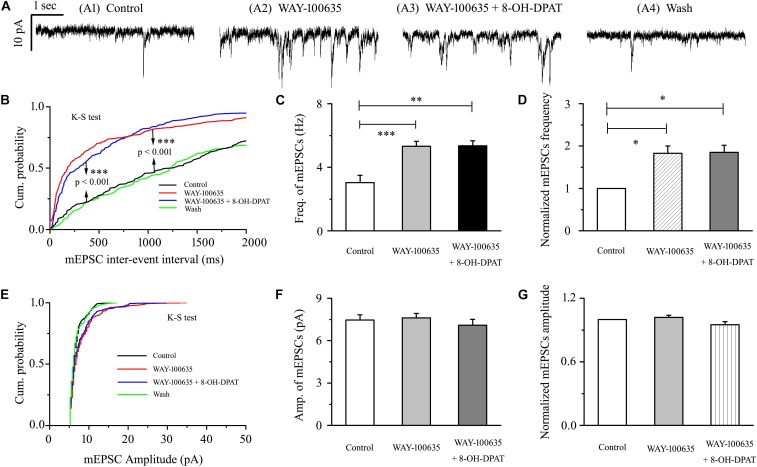
8-OH-DPAT-induced decreases in the frequency of mEPSCs in OFF-type RGCs were blocked by WAY-100635. **(A)** Representative recordings showing that WAY-100635 prevented 8-OH-DPAT-induced changes in mEPSC frequency. **(A1)** Control condition; **(A2)** during WAY-100635 treatment; **(A3)** during WAY-100635 + 8-OH-DPAT treatment; **(A4)** recovery. **(B,E)** Cumulative interevent interval and amplitude distributions of the mEPSCs in a representative neuron during control recording and during WAY-100635 and WAY-100635 + 8-OH-DPAT application. WAY-100635 and WAY-100635 + 8-OH-DPAT significantly shifted the distribution of interevent intervals to the left **(B)** but did not shift the distribution of mEPSC amplitudes **(E)**. The WAY-100635 and WAY-100635 + 8-OH-DPAT-induced changes in the distribution of interevent intervals and amplitudes were statistically significant (*n* = 8). ^∗∗∗^*p* < 0.001, Kolmogorov–Smirnov test. Summarized data for the frequency **(C)** and amplitude **(F)** of the mEPSCs (*n* = 9). **(D,G)** Normalized mEPSC frequency and amplitude (*n* = 9). ^∗^*p* < 0.05, ^∗∗^*p* < 0.01, and ^∗∗∗^ *p* < 0.001, ANOVA.

WAY-100635 alone significantly increased the baseline frequency, but not the amplitude, of mEPSCs (Figure 7A,B,E) in glaucomatous RGCs. The frequency increased from 3.03 ± 0.46 to 5.32 ± 0.31 Hz (*n* = 9, *p* < 0.001; Figure 7C), 183 ± 17% of the control level (*n* = 9, *p* < 0.05; Figure 7D). The amplitude did not decrease (7.46 ± 0.36 pA in the control group vs. 7.61 ± 0.31 pA in the treated group; *n* = 9, *p* > 0.05; Figure 7F), remaining at 102 ± 2% of the control level (*n* = 9, *p* > 0.05; Figure 7G). The effects of WAY-100635 and WAY-100635 + 8-OH-DPAT on the cumulative distributions of the interevent intervals and amplitudes of glutamatergic mEPSCs, as assessed by the Kolmogorov–Smirnov test, are shown in Figure 7B (*n* = 8, *p* < 0.001) and Figure 7E (*n* = 8, *p* > 0.05), respectively. WAY-100635 and WAY-100635 + 8-OH-DPAT markedly decreased the interevent interval, but not the amplitude, of mEPSCs relative to the control. After preincubation with WAY-100635 (10 μM), addition of 10 μM 8-OH-DPAT was unable to reduce the frequency (5.32 ± 0.46 Hz before vs. 5.36 ± 0.3 Hz after 8-OH-DPAT application; *n* = 9, *p* > 0.05; Figure 7C) of mEPSCs to 185 ± 17% of the WAY-100635 application (*n* = 9, *p* > 0.05; Figure 7D). In the presence of WAY-100635 (10 μM), 8-OH-DPAT (10 μM) did not significantly change the amplitude (7.46 ± 0.36 pA before vs. 7.1 ± 0.41 pA after 8-OH-DPAT application; *n* = 9, *p* > 0.05; Figure 7F) of the glutamatergic mEPSCs, which remained at 95 ± 3% of the control level (*n* = 9, *p* > 0.05; Figure 7G).

### 8-OH-DPAT Did Not Significantly Change the Amplitude of Glutamate-Induced Currents in Both OFF- and ON-Type RGCs in Glaucoma

**FIGURE 8:**
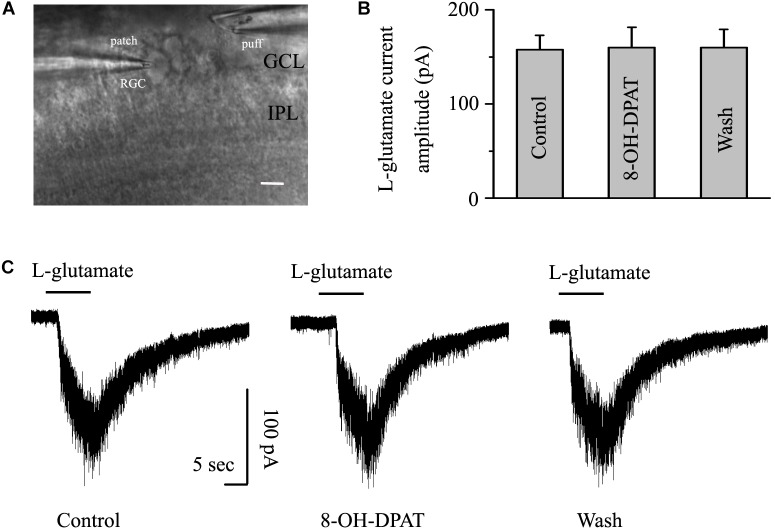
8-OH-DPAT did not change the amplitude of L-glutamate-induced current in OFF- and ON-type RGCs. The micrograph in **(A)** was taken with an infrared interferometric phase microscope, and the retinal layers can be seen. The microelectrode on the left was used for sealing the RGC, and the microelectrode on the right was used for pressure injection. **(B)** Bar graph illustrating no differences in the peak amplitudes of the responses recorded from control, 8-OH-DPAT and recovery conditions (*n* = 7). **(C)** Representative traces of L-glutamate-induced current during control (left), 8-OH-DPAT (middle), and wash (right) conditions. Vertical scale bar: 100 pA; horizontal scale bar: 5 s. GCL, ganglion cell layer; IPL, inner plexiform layer. Scale bar: 10 μm.

As shown in Figure 8A, another glass microelectrode with a tip diameter of 3–4 μm was placed approximately 50 μm away from the target cell soma. Pressure puffing L-glutamate onto RGCs elicits an inward current (Figure 8C); L-glutamate was puffed for 8 s through the pressure feeding system. After the entire baseline was stabilized for 5 min, gravity perfusion was conducted, followed by 8-OH-DPAT treatment for 6 min. We also tested the two types of RGCs in glaucoma. Since the OFF- and ON-type RGCs had the same responses to the glutamate-induced current and 8-OH-DPAT, we combined the data from the two types of RGCs in this section. However, the mean amplitude of L-glutamate-induced current was not significantly different between the control and the 8-OH-DPAT-treated groups (control: 157.52 ± 15.19 pA, 8-OH-DPAT: 159.73 ± 21.65 pA, wash: 160 ± 19.28 pA, *n* = 7, *p* > 0.05; Figure 8B). These results from patch-clamp recordings of RGCs suggested that 8-OH-DPAT caused no profound changes in the post-synaptic glutamate receptor properties under glaucoma conditions.

### 8-OH-DPAT Did Not Significantly Change the Frequency or Amplitude of Glutamatergic sEPSCs and mEPSCs in ON-Type RGCs in Glaucoma

**FIGURE 9:**
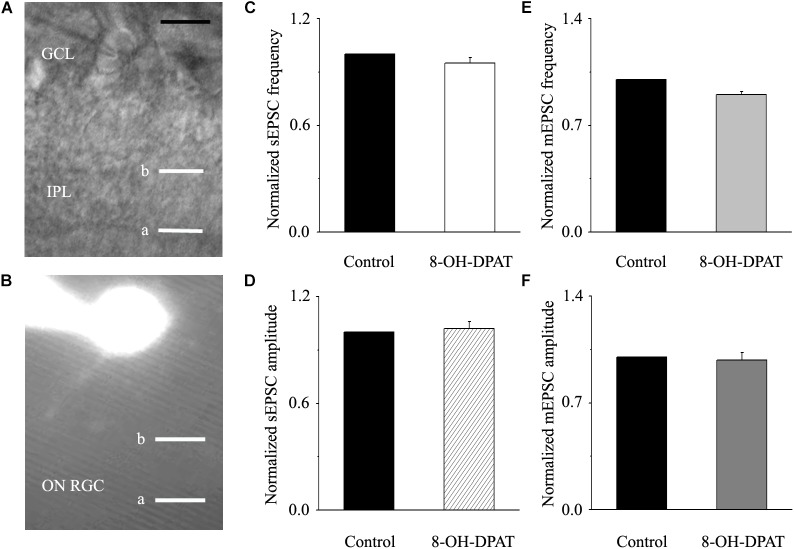
8-OH-DPAT did not change the frequency or amplitude of sEPSCs or mEPSCs in ON-type RGCs. **(A,B)** The micrograph in **(A)** was taken with an infrared interferometric phase microscope, and the retinal layers can be seen. Representative Lucifer yellow-filled ON-type RGCs with dendritic arborizations in the distal (b) parts of the IPL are shown. **(C,D)** Normalized sEPSC frequency and amplitude (*n* = 7). **(E,F)** Normalized mEPSC frequency and amplitude (*n* = 10). GCL, ganglion cell layer; IPL, inner plexiform layer. Scale bar: 10 μm. a, the dendritic arborizations in the proximal (a) parts of the IPL.

Morphologically, ON-type RGCs were characterized based on whether their dendrites terminated in the proximal (a) or distal (b) parts of the IPL (Figure 9A,B). 8-OH-DPAT (10 μM) had no effect on either the frequency or amplitude of glutamatergic sEPSCs in ON-type RGCs in glaucomatous RGCs. The frequency in treated RGCs was 95 ± 3% of the control level, and the amplitude was 102 ± 4% of the control level (*n* = 7, *p* > 0.05; Figure 9C,D). We also examined the effect of 8-OH-DPAT (10 μM) on glutamatergic mEPSCs in ON-type RGCs in the presence of TTX (1 μM). As before, 8-OH-DPAT did not significantly change either the frequency (90 ± 2% of the control level) or amplitude (98 ± 5% of the control level) of glutamatergic mEPSCs (*n* = 10, *p* > 0.05; Figure 9E,F).

## Discussion

There is currently no effective neuroprotective agent for the treatment of glaucoma. Therefore, on the basis of previous studies, we further studied the regulation of 8-OH-DPAT on glutamatergic synaptic transmission in chronic glaucomatous RGCs. First, we showed that intraocular hypertension upregulates GS and EAAT2 protein expression in the retina. Second, whole-cell patch-clamp recordings demonstrated that administration of 8-OH-DPAT significantly decreases the frequency, but not the amplitude, of glutamatergic sEPSC in OFF-type RGCs, with similar results for mEPSCs. Third, the modulatory effects of 8-OH-DPAT on EPSCs were completely blocked by the selective 5-HT1A receptor antagonist WAY-100635. Fourth, WAY-100635 alone markedly increased the baseline frequencies of sEPSCs and mEPSCs in OFF-type RGCs. Fifth, 8-OH-DPAT had no effect on the amplitude of the L-glutamate-induced post-synaptic current. Finally, the effects of 8-OH-DPAT at the synaptic level in OFF-type RGCs did not exist at all in ON-type RGCs. Altogether, the results of this study reveal a novel mechanism by which activation of the 5-HT1A receptor specifically modulates the neuronal glutamatergic system of OFF-type RGCs and thereby helps protect RGCs in glaucoma.

Glutamine synthetase, the most important enzyme in glial cells, is used to clear excess glutamate out of the cell and to maintain normal glutamate concentrations. Increased concentrations of glutamate in the vitreous cavity resulted in increased GS immunoreactivity in Müller cell end-feet *in vivo* (Shen et al., 2004). If cultured rat glial cells are exposed to a high concentration of glutamate in the extracellular environment, the immune activity of GS and its RNA level can also be upregulated (Shen et al., 2004). Therefore, based on previous works and research in our chronic glaucoma model, we confirmed that intraocular hypertension can upregulate GS protein expression.

Glutamate transporters are used to transport the most important excitatory neurotransmitter in the neurotransmitter transport family: glutamate. There are two main glutamate transporter families: the Na^+^-dependent EAAT family and the vesicular glutamate transporter (VGLUT) family. EAAT transports glutamate from the synaptic cleft and extrasynaptic sites into neurons and glial cells via a glutamate reuptake mechanism, while VGLUT transports glutamate into synaptic vesicles via the cell cytoplasm (Ishikawa, 2013). Five subtypes of EAAT have been cloned in mammals: EAAT1 (GLAST), EAAT2 (GLT-1), EAAT3 (EAAC1), EAAT4, and EAAT5 (Danbolt, 2001). EAAT2 is mainly expressed in glial cells in CNS and is the most important glutamate transporter in adult tissues (Rothstein et al., 1996). The loss of EAAT2 is involved in the development of CNS neurodegenerative diseases, such as Alzheimer’s disease and Huntington’s disease, and peripheral spinal cord motor neuropathies, such as amyotrophic lateral sclerosis (ALS) (Yi and Hazell, 2006). Chronic glaucoma is a degenerative disease of the CNS; thus, this study focused on EAAT2. When glutamate transporters are inhibited by drugs, neurons in the retina will be exposed to high concentrations of glutamate, thus causing severe excitatory toxicity damage (Izumi et al., 2002; Yanagisawa et al., 2015). It has been reported in many studies that EAAT2 expression is downregulated in neuron damage (Fontana, 2015), but our results show the opposite, that EAAT2 expression is upregulated in a chronic glaucoma rat model. These results might reflect a compensatory mechanism of the organism under the stimulation of pathological stress. A similar mechanism has been proposed in a study on EAAT3 (Bjorn-Yoshimoto and Underhill, 2016). In the murine model of Huntington’s disease, the upregulation of plasma membrane EAAT3 (Petr et al., 2013) was accompanied by a decrease in the expression of the cystine/Glu exchangers (Frederick et al., 2014). When cystine is downregulated, the internal transport system upregulates EAAT3 to maintain its reuptake balance, which is the precise compensatory mechanism in Huntington’s disease cell lines. Under our experimental conditions, GS expression was upregulated during glaucoma with a concomitant increase in EAAT2 expression; this finding may demonstrate a similar compensatory mechanism because we found that the frequency of glutamatergic post-synaptic currents in normal and glaucomatous RGCs was not significantly altered. We hypothesized that the purpose of increasing GS and EAAT2 in the pathological state of glaucoma was to maintain the metabolic balance of glutamic acid. Therefore, under the conditions of our experiment, the pathological factors of glaucoma did not affect neurotransmitter release. However, whether other related metabolites in glutamate metabolism are changed in glaucoma remains to be further explored in subsequent experiments.

There is also substantial evidence that serotonin itself is an important neurotransmitter in the retina (Hidaka, 2009). 8-OH-DPAT, as a 5-HT1A/7 receptor agonist, reduced evoked EPSC amplitude in wild-type and 5-HT1B knockout mice. Moreover, the effect of 8-OH-DPAT can be significantly blocked by the mixed 5-HT2/7 receptor antagonist ritanserin but can only be slightly weakened by the selective 5-HT1A receptor antagonist WAY-100635 (Smith et al., 2001). In the mammalian suprachiasmatic nucleus (SCNs), only activation of 5-HT7 receptors rather than the 5-HT1A receptors can reduce the amplitude of EPSCs induced by stimulation of the optic nerve (Smith et al., 2001). However, under our experimental conditions, the 8-OH-DPAT-mediated regulation of synaptic current was completely inhibited by the specific 5-HT1A receptor blocker WAY-100635. 8-OH-DPAT is an agonist that can act on both 5-HT1A and 5-HT7 receptors. Our finding suggests that in our chronic glaucoma model, the 5-HT1A receptor is the specific receptor involved in presynaptic regulation of glutamatergic afferent signaling. Furthermore, the density of GS and EAAT2 was significantly increased in glaucomatous retinas, but the high expression of GS induced by glaucoma was downregulated by 8-OH-DPAT, which led to the inhibition of glutamate release. The high expression of EAAT2 induced by glaucoma was downregulated by WAY-100635, while increasing the frequency of glutamatergic sEPSCs and mEPSCs.

In a glaucoma mouse model that was obstructed by micromagnetic bead injection, it was found that the visual function was impaired before any change in anatomical structure, and the OFF-type RGCs were more likely to be impaired than ON-type RGCs (Sabharwal et al., 2017). Many experiments have also shown that high IOP can lead to impaired OFF-type RGC function and anatomical dissection of OFF sublamina in the inner reticular layer (Della Santina et al., 2013; El-Danaf and Huberman, 2015; Ou et al., 2016). Sabharwal et al. (2017) found that reductions in light offset will increase the spike levels of OFF-type RGCs and will only reduce the sizes of the OFF-type RGC receptive field centers in the condition of photopic vision, but not for ON-type RGCs (Della Santina et al., 2013; Chen et al., 2015). In general, OFF-transient RGCs exhibit faster functional and structural vulnerability than other RGCs (Della Santina et al., 2013). Consistent with this observation, we found that activation of the 5-HT1A receptor specifically reduced excitatory glutamate synaptic transmission to OFF-type RGCs in chronic glaucoma.

In this case, we ask whether the site of action of the 5-HT1A receptor is presynaptic or post-synaptic. To answer this question, there are two theoretical experimental methods to verify. One approach is to examine the effect of 8-OH-DPAT on the post-synaptic current. If the frequency of the post-synaptic current is changed, then the effect is due to the release of presynaptic neurotransmitters; if the amplitude is changed, then the effect is due to the post-synaptic effect. Another method is to record the effect of 8-OH-DPAT on the post-synaptic current amplitude induced by a local direct puff L-glutamate. In our results, we found that 8-OH-DPAT reduced the frequency of EPSCs in both spontaneous and miniature of OFF-type RGCs but had no effect on the amplitude. The fact that the amplitude was not affected indicates that the regulation of 8-OH-DPAT mainly plays a presynaptic role and mediates the release of synaptic vesicles. The complete ineffectiveness of 8-OH-DPAT on the local pressure injection of L-glutamate-induced current further provides evidence that the presynaptic mechanism dominates in this experiment.

Given all of our findings, we propose that selective stimulation of retinal 5-HT1A receptors and the resulting inhibition of glutamate release by OFF-type RGCs may serve as a potential mechanism or target for future consideration in the neuroprotective diagnosis and treatment of glaucoma. Our findings also contribute to the current understanding of the synaptic effects and mechanisms of RGC injury.

## Ethics Statement

This study was carried out in accordance with the recommendations of “the Association for Research in Vision and Ophthalmology (ARVO) Statement for the Use of Animals in Ophthalmic and Vision Research and the guidelines of Fudan University on the ethical use of animals.” The protocol was approved by the Fudan University Committee.

## Author Contributions

XZ and JW designed the research. XZ and GL performed the research. SZ analyzed the data. XZ and SZ wrote the manuscript. JW modified the manuscript.

## Conflict of Interest Statement

The authors declare that the research was conducted in the absence of any commercial or financial relationships that could be construed as a potential conflict of interest.
