# Supplementary material for: 5-HT1A Receptor Agonist Promotes Retinal Ganglion Cell Function by Inhibiting OFF-Type Presynaptic Glutamatergic Activity in a Chronic Glaucoma Model
Source: Front Cell Neurosci. 2019 May 3;13:167. doi: 10.3389/fncel.2019.00167 (PMC6509153; doi:10.3389/fncel.2019.00167)
Supplement: Supplementary file 1 [file Data_Sheet_1.docx]

**5-HT1A receptor agonist promotes retinal ganglion cell function by inhibiting OFF-type presynaptic glutamatergic activity in a chronic glaucoma model**

Xujiao Zhou^a,b,c^, Gang Li^a,b,c^, Shenghai Zhang^a,b,c*^, Jihong Wu^a,b,c,d*^

^a^ Eye Institute, Eye & ENT Hospital, State Key Laboratory of Medical Neurobiology, Institutes of Brain Science and Collaborative Innovation Center for Brain Science, Shanghai Medical College, Fudan University, Shanghai 200032, China

^b^ Shanghai Key Laboratory of Visual Impairment and Restoration, Shanghai 200032, China

^c^ NHC Key Laboratory of Myopia (Fudan University); Key Laboratory of Myopia, Chinese Academy of Medical Sciences, Shanghai 200032, China

^d^ Department of Ophthalmology and Vision Science, Eye & ENT Hospital, Fudan University, Shanghai 200032, China

* Correspondence: [zsheent@163.com](mailto:zsheent@163.com) (S. Zhang) and [jihongwu@fudan.edu.cn](mailto:jihongwu@fudan.edu.cn) (J. Wu).

**Author contributions:** Xujiao Zhou and Jihong Wu designed the research, Xujiao Zhou and Gang Li performed the research, Shenghai Zhang analyzed the data, Xujiao Zhou and Shenghai Zhang wrote the paper, and Jihong Wu modified the paper.

**Running title:** 5-HT1A receptors modulate glutamate release

**Keywords:** 5-HT1A receptor, OFF-type RGCs, glaucoma, glutamate release, neuroprotection

**Supplementary materials:**


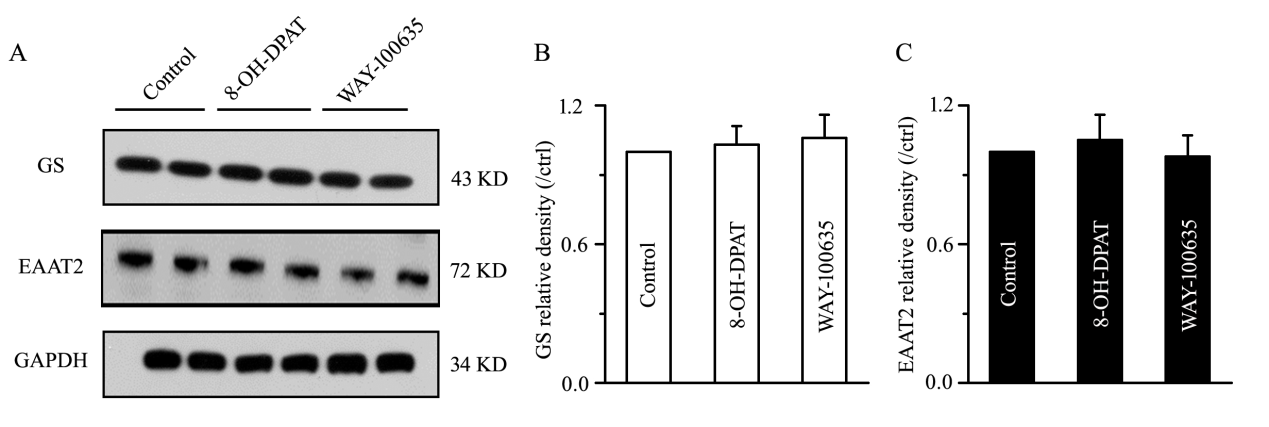


**Supplementary Figure S1. 8-OH-DPAT and WAY-100635 did not affect the protein expression levels of GS and EAAT2 in the normal retinas.** (A) Western blotting analysis of GS and EAAT2 protein expression levels in blank control, blank control + 8-OH-DPAT and blank control + WAY-100635. (B, C) Densitometric analysis of GS (B) and EAAT2 (C) in normal retinas (n = 6). GS and EAAT2 expression levels were normalized to the expression in the control retinas.
